# Fumonisin B_1_ Interaction with Mg-Al and Mg-Fe Layered Double Hydroxides: Removal Efficiency and Mechanisms

**DOI:** 10.3390/ma13194344

**Published:** 2020-09-29

**Authors:** Jakub Matusik, Youjun Deng

**Affiliations:** 1 Faculty of Geology, Geophysics and Environmental Protection; Department of Mineralogy, Petrography and Geochemistry, AGH University of Science and Technology, al. Mickiewicza 30, 30-059 Krakow, Poland; 2 Department of Soil and Crop Sciences, Texas A&M University, College Station, TX 77843-2474, USA; yjd@tamu.edu

**Keywords:** layered double hydroxides, adsorption, fumonisin B1, carbonates, XPS analysis

## Abstract

Mycotoxins in feed and food are highly toxic and pose a serious danger even at very low concentrations. The use of bentonites in animal diet can reduce toxin bioavailability. However, some mycotoxins like fumonisin B1 (FB1) form anionic species which excludes the use of negatively charged clays. Layered double hydroxides (LDH) with anion-exchange properties, in theory, can be perfect candidates to adsorb FB1. However, fundamental research on the use of LDH for mycotoxins removal is scarce and incomplete. Thus, the presented study was designed to explore such a possibility. The LDH materials with differing chemistry and layer charge were synthesized by co-precipitation both from metal nitrates and chlorides and were then tested for FB1 removal. XRD, FTIR, XPS, and chemical analysis were used for the LDH characterization and to obtain insight into the removal mechanisms. A higher adsorption capacity was observed for the Mg/Al LDH samples (~0.08–0.15 mol/kg) in comparison to the Mg/Fe LDH samples (~0.05–0.09 mol/kg) with no difference in removal efficiency between Cl and NO_3_ intercalated LDH. The adsorption capacity increased along with lower layer charge of Mg/Al and was attributed to the lower content of bonded carbonates and the increase of non-polar sites which led to matching between the adsorption domains of LDH with FB1. The FTIR analysis confirmed the negative effect of carbonates which hampered the adsorption at pH 7 and led to the highest adsorption at pH 5 (FB1 content ~15.8 ± 0.75 wt.%). The fast surface adsorption (1–2 min) was dominant and XRD analysis of the basal spacing indicated that no FB1 intercalation occurred in the LDH. The XPS confirmed a strong interaction of FB1 with Mg sites of LDH at pH 5 where the interaction with FB1 carboxylate moieties COO^−^ was confirmed. The research confirmed a high affinity and selectivity of LDH structures towards anionic forms of FB1 mycotoxin.

## 1. Introduction

Mycotoxins are complex organic compounds produced as a result of metabolic processes by filamentous fungi of different genera, e.g., *Fusarium*, *Aspergillus* and *Penicillium*. Currently, there are about 300 known metabolites which have adverse effects on living organisms including animals and humans [1]. The most important classes of mycotoxins that are unwanted in food and feed products are aflatoxins, trichothecenes, zearalenone, ochratoxins, and fumonisins. Most hazardous mycotoxins pose a serious danger even at very low concentrations and have been reported to be highly toxic and carcinogenic [2].

The contamination of food and feed by mycotoxins is a serious global problem that is responsible for significant economic losses. It is estimated that approximately 25% of all crops worldwide contain mycotoxins [1]. The mycotoxins presence is due to fungal infection of crops, particularly observed for cereals, e.g., maize, barley, wheat, rye, and oat. This causes health problems including chronic diseases leading to liver disorders, immunologic effects, digestive disorders, reproductive and metabolic disorders, and even death. The adverse effects of mycotoxins and their negative impact on the food market has led to the development of different prevention and decontamination strategies [1,3,4]. One category of approaches includes physical methods (e.g., sorting, separation, washing, or irradiation) and chemical treatment (the use of bases and oxidizing agents). The other category involves decontamination using adsorbents which could be potentially included in the animal diet. Such materials having adsorption properties can reduce toxin bioavailability through their immobilization. These include micro- and nano-sized minerals and non-mineral particles, e.g., clay minerals, zeolites, and activated carbons and their derivatives. Currently, bentonites, which are abundant sedimentary rocks rich in smectite clay minerals, have demonstrated a protective role against aflatoxins [5,6,7,8,9]. However, some mycotoxins are known to form anionic species, e.g., fumonisin B_1_ (FB1) which cannot be efficiently removed by the materials listed above due to their exclusive cation exchange properties. FB1 has a structure containing both basic –NH_2_ and acidic –COOH functional groups. Its charge changes as a function of solution pH and is mostly negative due to –COOH deprotonation. Therefore, in this study for its removal, a different class of materials was tested, i.e., layered double hydroxides (LDHs), which are also applied in catalysis, polymer chemistry, biomedicine, and wastewater treatment [10,11,12,13,14,15,16].

LDHs are crystalline phases classified as non-silicate oxides and hydroxides of the following general chemical formula: [M^II^_1−x_M^III^_x_(OH)_2_]^X+^[(Am^−^)_x/m_·nH_2_O]^X−^ [17]. The first part of the formula represents a positively charged brucite-like layer containing both di- and trivalent cations (M^II^ and M^III^), while the second part shows the interlayer composition where hydrated anions are present. The LDHs show evident similarities to natural clay minerals. These include a layered structure, diverse chemical composition due to isomorphous substitutions, variable layer charge, and rheological and colloidal properties. However, the main difference is their ability to exchange anions in contrast to clay minerals [18]. The brucite-like layer can have varied chemistry; the most common M^II^ cations are Mg, Zn, Mn, Co, and Ni, and the M^III^ cations are Al, Fe, and Cr. In turn, the layer charge is most compensated by the following anions: CO_3_^2−^, Cl^−^, NO_3_^−^, and SO_4_^2−^. These anions control the interlayer distance, water content, and subsequently influence the adsorption properties. For applications, the LDH particles are most often synthesized using aqueous co-precipitation technique and thus it is possible to easily control their chemistry [19]. All these benefits allow for the adjustment of the LDH adsorbents for a specific target, in this case the FB1 mycotoxin.

Thus far, reports have been published on the adsorptive removal of FB1 by surfactant-treated minerals, e.g., clinoptilolite [20,21]. To the best of our knowledge, to date, research on the use of LDH for mycotoxins has not been published. Therefore, the objective of the presented study was to evaluate the adsorption properties of chemically different LDH materials in reaction with FB1 in model aqueous solutions. For the first time, this research investigates the mechanisms and factors which affect the removal efficiency by applying several analytical methods including X-ray diffraction, FTIR spectroscopy, and X-ray photoelectron spectroscopy.

## 2. Experimental Methods

### 2.1. Materials

**Figure 1:**
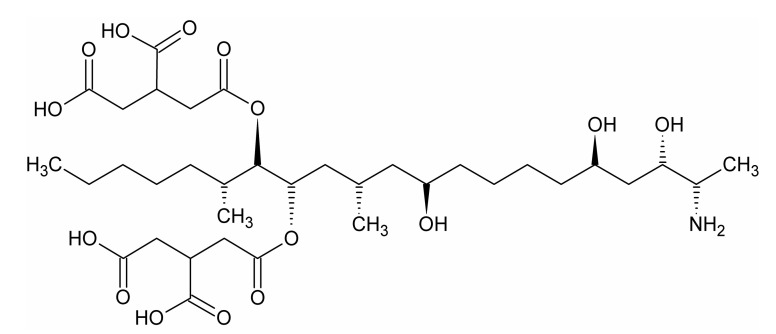
Structure of fumonisin B_1_ molecule.

The following chemicals of analytical grade were used for the LDH synthesis: MgCl_2_·6H_2_O, Mg(NO_3_)_2_·6H_2_O, AlCl_3_·6H_2_O, AlNO_3_·9H_2_O, FeCl_3_·6H_2_O, Fe(NO_3_)_3_·9H_2_O, and NaOH. The chemicals were obtained from Avantor Company (Gliwice, Poland). For the adsorption experiments, a fumonisin B_1_ from *Fusarium moniliforme* (FB1) was purchased from Sigma-Aldrich (CAS: 116355-83-0, ≥98% HPLC, St. Louis, MO, USA) (Figure 1). The following chemicals for FB1 quantification were obtained from Sigma-Aldrich: *o*-phthaldialdehyde, methanol, acetonitrile, disodium tetraborate, and 2-mercaptoethanol. Deionized water was used in all the experiments, unless stated otherwise.

### 2.2. Synthesis of LDH

In total, 12 LDH materials were prepared as listed in Table 1. The materials differed in the layer chemistry (Mg/Al or Mg/Fe), metal ratio (2:1, 3:1, 4:1, 6:1 and 8:1), and type of interlayer anion (Cl^−^ or NO_3_). The materials were synthesized by the standard co-precipitation method using aqueous metal salts. The solution containing both M(II) and M(III) in stoichiometric amounts was prepared with 100 mL of DI water. Regardless of the M(II) to M(III) ratio, the sum of the chemicals’ mass was set at a constant. Thus, the solution density was also constant. This solution was slowly mixed with aqueous 5 M NaOH in a 2 L beaker which initially contained 400 mL of water at pH 10. The precipitate formed in the 2 L beaker was constantly mixed with magnetic stirrer at room temperature. The whole synthesis lasted about 30–40 min. The portions of metal solution and base solution were gradually injected to maintain a constant pH 10 throughout the synthesis. In the end, the formed precipitates were aged for 2 h at room temperature (22 °C) and were then washed with water and dried at 70 °C for 24 h. The materials with 6:1 and 8:1 metal ratios were not aged to prevent the formation of metal hydroxides (e.g., brucite) as indicated in our previous trials.

### 2.3. Adsorption Experiments

The experiments were designed to investigate the adsorption efficiency and mechanisms in the reaction of FB1 with different LDH materials. All experiments were run in duplicates. The 200 ppm stock solution of FB1 was prepared in 1:1 acetonitrile/water solution. In all the experiments, LDH was introduced into the FB1 solution in the form of suspension. The LDH suspensions (2 mg/mL) were prepared by careful dispersion of the LDHs followed by sonification to ensure their homogeneity.

The first experiment performed for all materials tested the efficiency in reaction with 8 ppm FB1 (pH_in_ 6.4). The LDH suspension containing 0.1 mg of the material (50 µL) was injected into 15 mL vials containing 5 mL of 8 ppm FB1 (suspension density: 20 mg/L). The suspensions were shaken for 24 h to achieve equilibrium. The second experiment tested the adsorption efficiency for an FB1 concentration of 1.0–8.0 ppm. The rest of the experimental conditions remained as in the first experiment. This enabled us to obtain the adsorption isotherms for the selected LDH materials. In the third experiment, the pH effect was studied in the range of 2.2–11.2. The 8 ppm FB1 solutions (2.5 mL) were diluted with 2.5 mL of an aqueous solution of HCl or NaOH in order to stabilize the pH to the required values. After the dilution, the starting FB1 concentration was equal to 4 ppm. The rest of the conditions remained as in the first experiment. The fourth and fifth experiments studied the effect of adsorbent dose and time on the removal efficiency, respectively. The following values of adsorbent dose were applied: 20, 40, 60, 80, 100, and 200 mg/L. In turn, the kinetic experiment was run in the time range from 0.5 to 180 min. In both experiments, the FB1 initial concentration was set to 4 ppm with the rest of conditions remaining the same as in the first experiment.

In all cases, the samples for the analyses were filtered with 0.2 µm syringe filters. The FB1 concentration was quantified using the Synergy HT Multi-Detection Microplate Reader (BioTek) equipped with a fluorescence detector. The excitation and emission wavelengths were set to 360 nm and 460 nm, respectively. Each sample was analyzed at least 4 times to ensure high accuracy. Prior to the measurements, the 200 µL aliquots of the analyzed samples were derivatized with 50 µL of *o*-phthaldialdehyde (OPA) reagent directly in the dedicated black microplates. The whole process of OPA injection and measurements were carried out automatically to ensure repeatability of the results. The OPA reagent was prepared by dissolving 80 mg of OPA in 2 mL of methanol and 10 mL of 0.1 M disodium tetraborate. Then, 100 µL of 2-mercaptoethanol was added and the solution was thoroughly mixed. The OPA reagent was prepared fresh before measurements and it was later stored in a vial wrapped with aluminum foil for no more than one week.

The reported adsorption capacity (A) was calculated by using the following formula:

A = (C_in_ − C_eq_)·V/m (1)

where C_in_ and C_eq_—initial and equilibrium concentration (mmol/L), respectively; V—solution volume (L), and m—adsorbent mass (kg).

### 2.4. Analytical Methods for Solid Samples

The solid materials were characterized by X-ray diffraction (XRD), Fourier transformed infrared (FTIR) spectroscopy, scanning (SEM) and transmission (TEM) electron microscopy, X-ray photoelectron spectroscopy (XPS), and particle size analyzer (PSD). Moreover, the chemical composition of the brucite-like sheet was determined by the classical wet method. For that, the materials were dissolved in nitric acid and the Mg(II), Al(III), and Fe(III) concentrations were measured with an atomic absorption spectroscopy (AAS)—GBC SavanthAA spectrometer (Braeside, Australia). The XRD patterns were acquired on a Rigaku Miniflex 600 (Tokyo, Japan) and Bruker D8 Advance (Billerica, MA, USA) diffractometer with CuKα (λ = 1.5418 Å) radiation. The patterns of powdered samples were recorded in the range of 2–72° 2θ with a 0.05° 2θ step. The infrared spectra were measured by KBr pellet technique (1 mg sample mixed with 200 mg KBr) with a Nicolet 6700 spectrometer (Thermo Scientific, Waltham, MA, USA). For each measurement, 64 scans were collected in the range of 4000–400 cm^−1^ with a 4 cm^−1^ resolution. The samples after FB1 adsorption experiments were recorded on a PerkinElmer Spectrum 100 spectrometer (Waltham, MA, USA) in transmission mode (range 4000–850 cm^−1^; resolution 4 cm^−1^). In this case, the samples’ suspensions were dried on a ZnS disc and placed in a dedicated holder. The SEM images were obtained using FEI Quanta 600 FEG microscope (Hillsboro, OR, USA) under low vacuum. The powdered samples were prepared by placing powdered material on a carbon tape. The TEM images were collected using FEI Tecnai G2 F20 microscope at 200 keV. The samples were prepared by drying a drop of diluted LDH suspension on a lacey formvar/carbon TEM grid. The X-ray photoelectron spectroscopy (XPS) was used for the characterization of selected samples after reaction with FB1. The samples, prepared in the form of suspensions, were dried of glass disc directly used for the analysis. The spectra were recorded by the Omicron XPS/UPS system (Uppsala, Sweden) with an Argus detector equipped with dual Mg/Al X-ray source (Materials Characterization Facility, Texas A&M University, College Station, TX, USA). The particle size distribution of the starting LDH materials was determined by a Beckman Coulter LS230 instrument (Brea, CA, USA) using diluted aqueous LDH suspensions.

## 3. Results and Discussion

### 3.1. Characterization of Adsorbents

#### 3.1.1. XRD Results and Chemistry of Brucite-Like Layers

**Figure 2:**
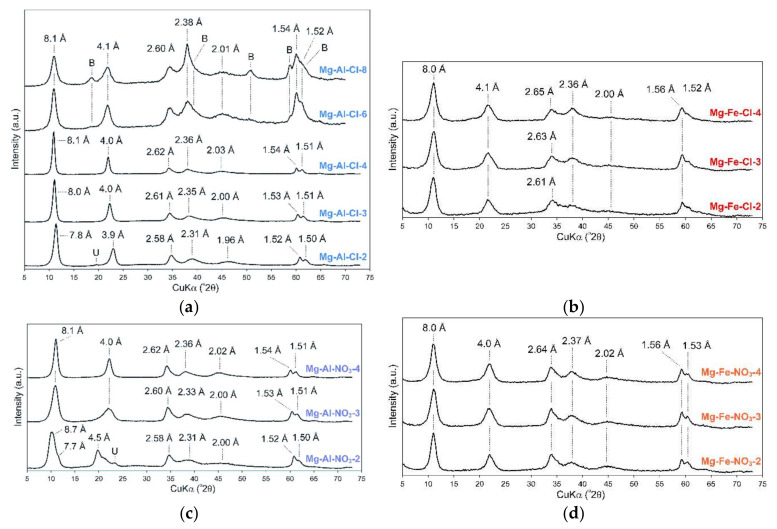
XRD patterns of raw LDH materials: (**a**) Mg-Al-Cl, (**b**) Mg-Fe-Cl, (**c**) Mg-Al-NO_3_, and (**d**) Mg-Fe-NO_3_, (B—brucite, U—unassigned).

The recorded XRD patterns confirmed the formation of LDH materials in all cases (Figure 2). The diffractograms were typical of a LDH structure with the basal reflections found in the 7.8–8.7 Å range depending on the materials’ chemistry [22]. The d_003_ values were also typical for LDH materials intercalated with chlorides, nitrates, and competitive carbonates from the air atmosphere. As shown in Table 1, the determined M(II)/M(III) ratio increased as expected. However, in some cases the values were lower than the expected values. In particular, this was observed for the samples with high Mg(II) contents. Nevertheless, the obtained materials had a clear difference in the M(II)/M(III) ratios and subsequently they significantly differed in their layer charge densities.

The peaks, due to other crystalline admixtures, were not observed, indicating the high purity of the LDH materials. The exception for that was the Mg-Al-Cl-8 sample where brucite was formed due to the high concentration of Mg(II) used in the synthesis (Figure 2a). For the Mg-Al-Cl samples, the basal spacing clearly increased with the increase of Mg/Al ratios from 7.8 to 8.1 Å (Figure 2a). This agreed with previous studies showing that the decrease of the layer charge leads to an increase of the interlayer spacing in the case of Cl-intercalated LDH materials [23]. This is due to a decrease in electrostatic attraction between positively charged layers and interlayer anions. In turn, for the Mg-Al-NO_3_ materials, the highest basal spacing value of 8.7 Å was noticed for the Mg-Al-NO_3_-2 sample. For the Mg-Al-NO_3_-3 and Mg-Al-NO_3_-4 samples, the d value dropped to 8.1 Å. This agrees with earlier studies showing that nitrate orientation in contrast to chloride is significantly affected by the layer charge density [24,25]. Its vertical position is observed for high charge materials; thus, the d values are higher than for LDH structures with lower charge where NO_3_ adopts a horizontal arrangement. Moreover, for the Mg-Al-NO_3_-2 sample a dual nature of the d_003_ peak was observed which shows that part of the layers were intercalated by NO_3_ (8.7 Å) and part by CO_3_^2−^ (7.7 Å) (Figure 2c). The changes in d values for the Mg-Fe-Cl and Mg-Fe-NO_3_ materials were not observed despite the clear differences in the brucite-like layer chemistry that were noticed. The values in all cases were equal to 8.0 Å (Figure 2b,d).

#### 3.1.2. FTIR Results

**Figure 3:**
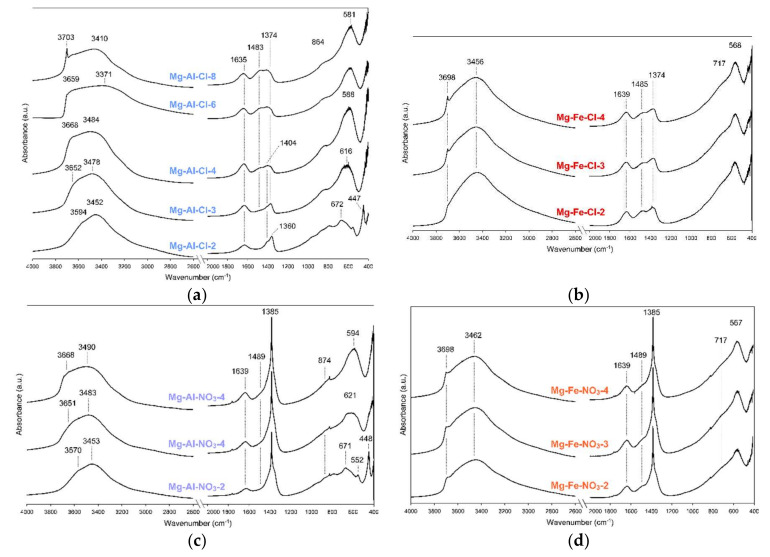
FTIR spectra of raw LDH materials: (**a**) Mg-Al-Cl, (**b**) Mg-Fe-Cl, (**c**) Mg-Al-NO_3_ and (**d**) Mg-Fe-NO_3_.

The FTIR spectra confirm and complement the data discussed in Section 3.1.1. The spectra are typical for hydrated LDH structure intercalated by chlorides or nitrates and accompanying carbonates. For the Mg-Al-Cl and Mg-Fe-Cl samples, the carbonate bands were found in the 1500-1350 cm^−1^ region (Figure 3a,b).

Two bands can be distinguished in this region: the 1374 cm^−1^ band attributed to monodentate carbonates and the 1483 cm^−1^ band attributed to bicarbonates [26]. It can be observed for the Mg-Al-Cl samples that the relative intensity of these bands visibly changes, indicating a greater proportion of bicarbonates with the decrease of layer charge. A similar trend was described in previous reports [19]. Such a trend was not evident for the Mg-Fe-Cl materials, where the intensity of the 1374 cm^−1^ and 1485 cm^−1^ bands remained similar despite chemistry differences. The presence of intercalated NO_3_ for the Mg-Al-NO_3_ and Mg-Fe-NO_3_ samples was evidenced by a sharp band at 1385 cm^−1^ attributed to N-O stretching vibrations (Figure 3c,d) [24]. However, the presence of the accompanying bicarbonates still cannot be excluded as the characteristic band is visible at 1489 cm^−1^. The formation of brucite was confirmed for the Mg-Al-Cl-8 sample rich in Mg(II) where a characteristic band at 3703 cm^−1^ was noticed (Figure 3a). A small content of brucite was also attested for the Mg-Fe-Cl and Mg-Fe-NO_3_ materials although this phase was not detected by XRD (Figure 3b,d). All the LDH samples contained water, thus a broad band in the 3800–3200 cm^−1^ region and a band at ~1640 cm^−1^ were observed. The position of this band did not change with the change in layer chemistry for the Mg-Fe materials (Figure 3b,d). In turn, a visible shift of this broad band towards higher wavenumbers was noticed along with the increase in Mg(II) content and subsequent decrease of layer charge for the Mg-Al materials (Figure 3a,b) [27]. A similar trend can be observed for the structural Me-O-Me bands which appear in the region below 800 cm^−1^. Regardless of the interlayer anions, for the Mg-Al samples a shift of main structural band was noticed from 672 to 581 cm^−1^ (Mg-Al-Cl samples) and from 671 to 594 cm^−1^ (Mg-Al-NO_3_).

#### 3.1.3. SEM/TEM Results

**Figure 4:**
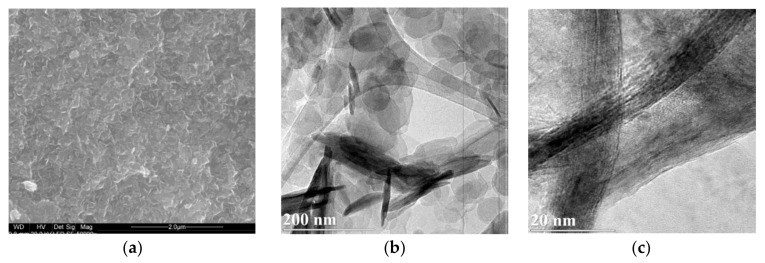

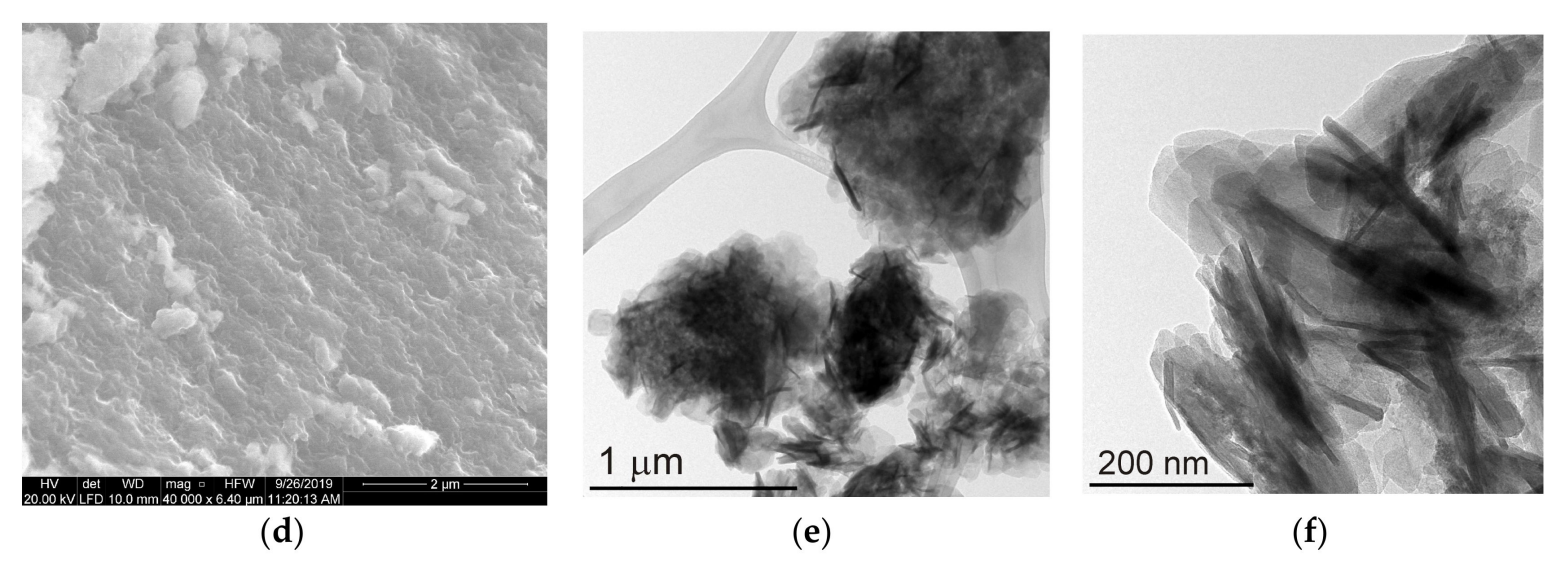
SEM and TEM images of LDH materials: (**a**–**c**) Mg-Al-Cl-2 and (**d**–**f**) Mg-Fe-Cl-2.

The microscopic observations did not reveal significant differences between Mg-Al and Mg-Fe samples. Regardless of the brucite-layer chemistry and the type of interlayer anion, the differences were hardly visible. Thus, the images of two representative Mg-Al-Cl-2 and Mg-Fe-Cl-2 samples were included. The SEM images showed aggregates of typical layered particles which started to be noticeable under magnification of 40,000–50,000× (Figure 4a,d). The sheet-like particles formed characteristic “house of cards” structures resulting from face to edge interaction. A TEM inspection enabled us to observe individual rounded particles with diameters in the range of ~20–200 nm and thickness in the range from ~10–20 nm. The observations at high magnification of ~400,000× showed nanometer-sized individual layers of the LDH particles (Figure 4c). The microscopic data agreed with particle size measurements (Figure S1) where two dominating populations of particles can be distinguished: (1) <250 nm—individual particles, and (2) 1.0–2.5 µm—aggregates of particles forming clusters.

### 3.2. Adsorption Experiments

**Figure 5:**
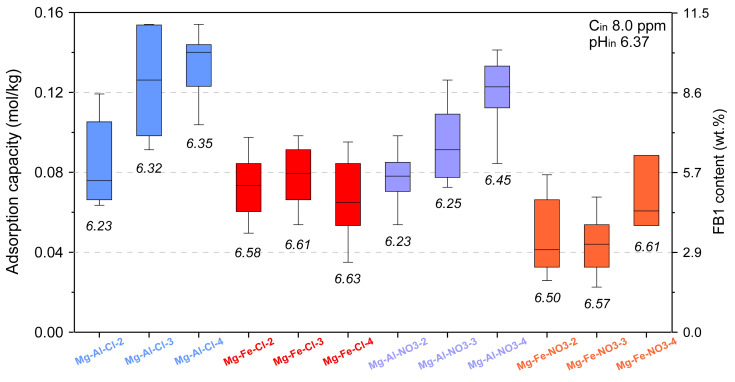
Adsorption capacity of LDH materials tested for 8 ppm FB1.

The initial test evaluating the LDH removal efficiency of FB1 was performed in identical conditions for all materials with the exception of Mg-Al-Cl-6 and Mg-Al-Cl-8 (Figure 5). In this experiment, the mass of introduced FB1 in relation to adsorbent mass was kept equal to 40%. This enabled the direct comparison of the adsorption capacity between all the materials. The results clearly show two groups of materials with different adsorption behaviors. The first group of Mg-Al materials intercalated either with Cl or NO_3_, which had a higher adsorption capacity for FB1. For this group, a trend of increasing FB1 adsorption capacity along with layer charge decrease could be noticed. The second group consisted of Mg-Fe materials with lower FB1 adsorption capacity, especially in the case of Mg-Fe-NO_3_ materials. For the Mg-Fe sample, the trend between adsorption capacity and layer charge was not evident. The pH_in_ of 6.37 did not change substantially after the experiments although visibly higher values were noticed for the Mg-Fe samples as compared to Mg-Al samples. The calculated average values of adsorption capacity (in mmol/kg) were as follows: Mg-Al-Cl-2: 0.083 ± 0.02, Mg-Al-Cl-3: 0.125 ± 0.027, Mg-Al-Cl-4: 0.134 ± 0.015, Mg-Fe-Cl-2: 0.072 ± 0.014, Mg-Fe-Cl-3: 0.080 ± 0.015, Mg-Fe-Cl-4: 0.068 ± 0.018, Mg-Al-NO_3_-2: 0.077 ± 0.015, Mg-Al-NO_3_-3: 0.093 ± 0.019, Mg-Al-NO_3_-4: 0.119 ± 0.017, Mg-Fe-NO_3_-2: 0.046 ± 0.016, Mg-Fe-NO_3_-3: 0.045 ± 0.016, and Mg-Fe-NO_3_-4: 0.066 ± 0.016. It is worth noting that the corresponding average FB1 content (presented in Figure 5) was in the range of ~3.0–10.0 wt.% with the highest values calculated for the Mg-Al samples. 

**Figure 6:**
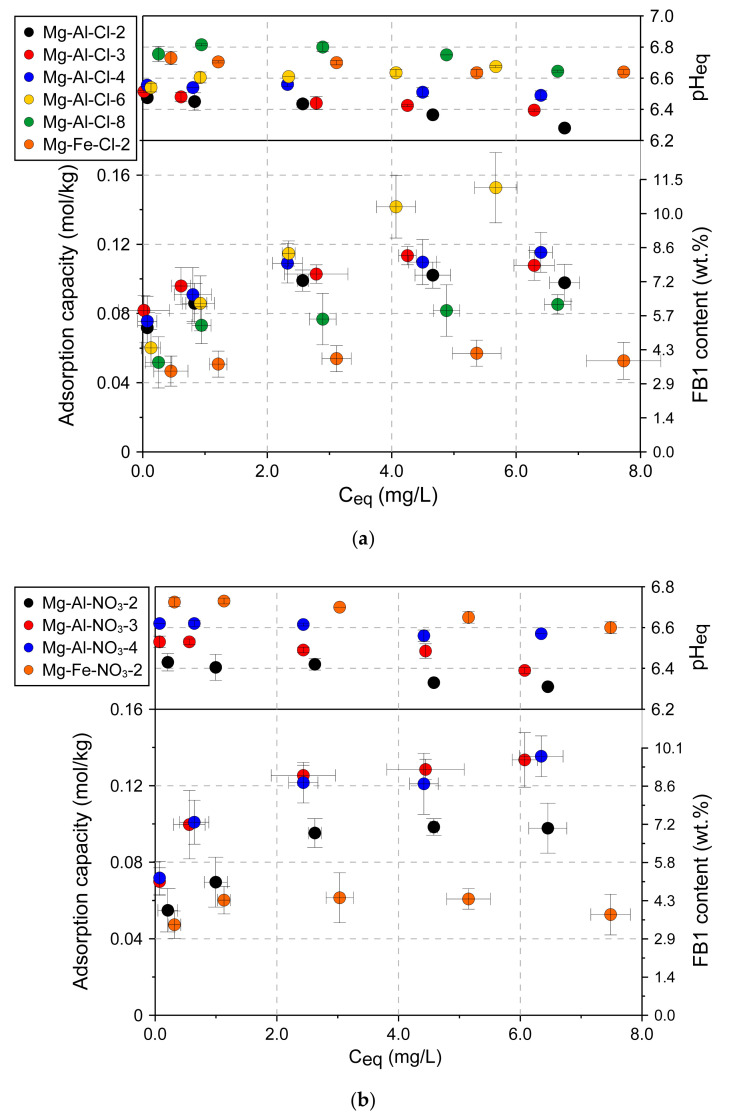
Adsorption isotherms for LDH materials: (**a**) Cl intercalated samples and (**b**) NO_3_ intercalated samples.

To confirm the trends observed in Figure 5 for the selected materials, full adsorption isotherms were determined in a broad FB1 concentration range (Figure 6). In agreement with the initial observations, the Mg-Fe materials showed visibly lower adsorption capacity: Mg-Fe-Cl-2: 0.047±0.009 mmol/kg and Mg-Fe-NO_3_-2: 0.047 ± 0.007 mmol/kg. Thus, these materials were excluded from further investigations. The full isotherms confirmed a trend of increasing adsorption capacity along with decreasing layer charge density for the following groups of materials: Mg-Al-Cl-2 < Mg-Al-Cl-3 < Mg-Al-Cl-4 < Mg-Al-Cl-6 (Figure 6a) and Mg-Al-NO_3_-2 < Mg-Al-NO_3_-3 < Mg-Al-NO_3_-4 (Figure 6b). In particular, the adsorption capacity of Mg-Al-Cl-6 sample with the lowest layer charge density had the greatest adsorption capacity of 0.153 ± 0.02 mmol/kg. Likely due to the high content of brucite as detected by XRD and FTIR in the Mg-Al-Cl-8 sample, its FB1 adsorption capacity was lower and did not follow the trend.

**Figure 7:**
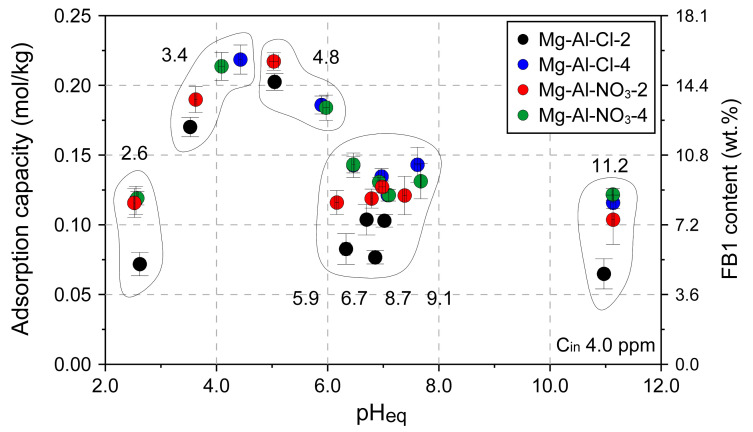
The pH effect on adsorption capacity of selected LDH materials. The values on the graph indicate initial pH values.

The pH effect was investigated for 4 selected materials: Mg-Al-Cl-2, Mg-Al-Cl-4, Mg-Al-NO_3_-2, and Mg-Al-NO_3_-4 (Figure 7). The results showed a significant impact of pH on the adsorption capacity. In general, similar trends for all the materials were observed with the FB1 greatest removal efficiency in the range of pH equilibrium equal to 4–5. In this range, the adsorption capacity reached 0.219 ± 0.01 mmol/kg which corresponds to 15.8 ± 0.75 wt.% of FB1 (Mg-Al-Cl-4 sample). The FB1 uptake was slightly lower in extreme acidic (pH < 3) or basic (pH > 11) conditions as compared to neutral pH values in the range of 6–8.

**Figure 8:**
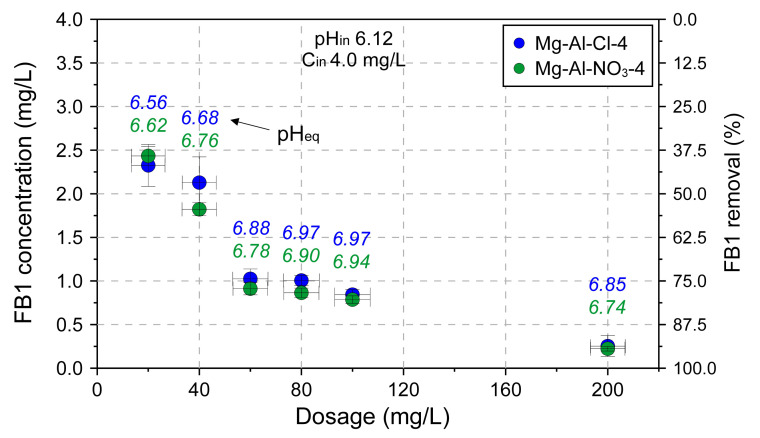
The dosage effect on adsorption capacity of selected LDH materials.

**Figure 9:**
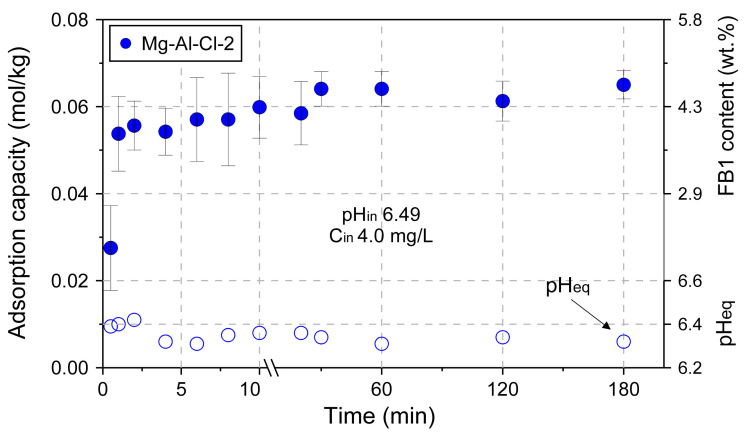
The adsorption kinetics examined for the Mg-Al-Cl-2 sample.

The effect of adsorbent dose on adsorption capacity was tested for the Mg-Al-Cl-4 and Mg-Al-NO_3_-4 samples (Figure 8). The experiment confirmed the remarkable adsorption properties of LDH towards FB1. The FB1 was removed in ~95% when the amount of introduced LDH was only 200 mg/L. The adsorption itself was a fast process with most of FB1 removed in the first minute of interaction, as shown for the Mg-Al-Cl-2 sample (Figure 9).

It is worth underlining that the obtained values of adsorption capacity were much greater than the values reported thus far. For example, a zeolitic material modified with octadecyldimethylbenzylammonium surfactant reached an adsorption capacity corresponding to 0.0149 mol/kg (10.819 mg/g) at pH 3 [21]. Surfactant-modified minerals were able to reduce FB1 concentration in acidic conditions with a visible decrease in removal efficiency with pH increase [20]. The other disadvantage of these materials is the much higher amount of adsorbent necessary for the reaction 0.4–10 g/L. The FB1 uptake was highly dependent on the surface coverage of the surfactant which determined the type of interactions changing from pH-dependent to hydrophobic pH-independent attraction [20].

### 3.3. Insight into Removal Mechanisms

#### 3.3.1. XRD and FTIR Analysis

The experimental results suggested that the interaction of FB1 molecule with LDH structure was complex and mainly influenced by the pH of the solution. This relates to the structure of FB1 having four distinct carboxyl groups which can undergo protonation/deprotonation and its possible conformational changes [28,29]. On the other side the LDH adsorbent structure shows different changes in stability due to pH, with partial dissolution occurring below pH 3 and high stability in basic conditions (pH 8–10) [30].

**Figure 10:**
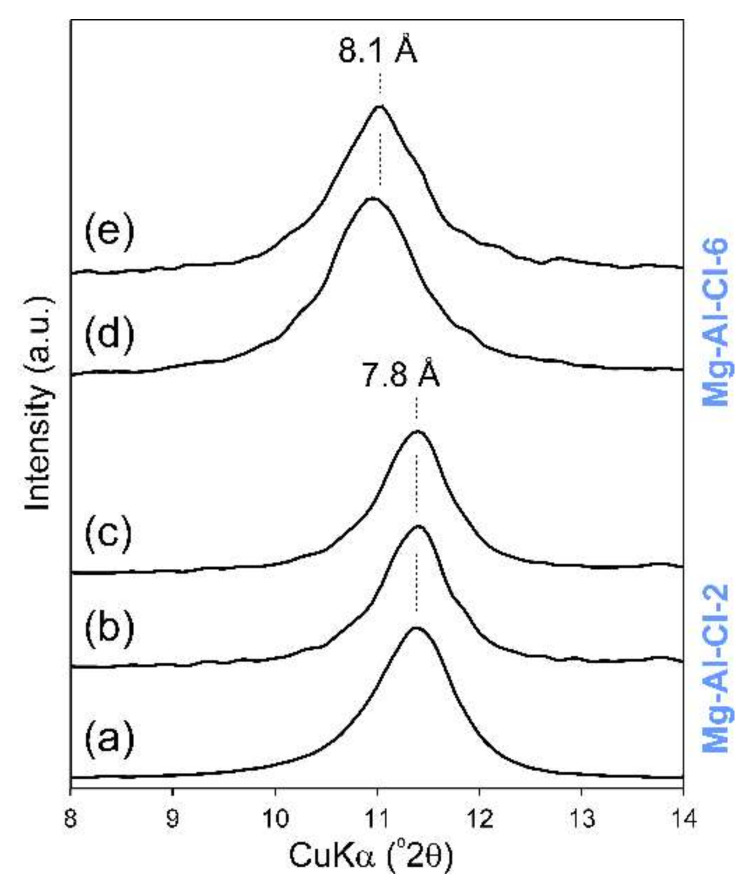
XRD patterns of selected LDH samples after reaction with FB1: (**a**) raw Mg-Al-Cl-2 sample, (**b**) FB1-treated Mg-Al-Cl-2 sample (pH 5, C_FB1_ 100 ppm), (**c**) FB1-treated Mg-Al-Cl-2 sample (pH 7, C_FB1_ 100 ppm), (**d**) raw Mg-Al-Cl-6 sample, and (**e**) FB1-treated Mg-Al-Cl-6 sample (pH 5, C_FB1_ 200 ppm).

Moreover, in the case of adsorbents having a layered structure, two main possibilities exist in terms of the location of adsorbate molecules. The first one is the migration of molecules into the interlayer space leading to intercalation. The second one is the surface adsorption of molecules. The research we performed showed that the intercalation did not take place in any of the applied conditions even at a very high concentration of FB1 equal to 100 or 200 ppm (Figure 10). The XRD results clearly showed that the initial basal spacing peak was not shifted and was still present at 7.8 Å (Mg-Al-Cl-2 sample) or 8.1 Å (Mg-Al-Cl-6 sample) (Figure 10).

**Figure 11:**
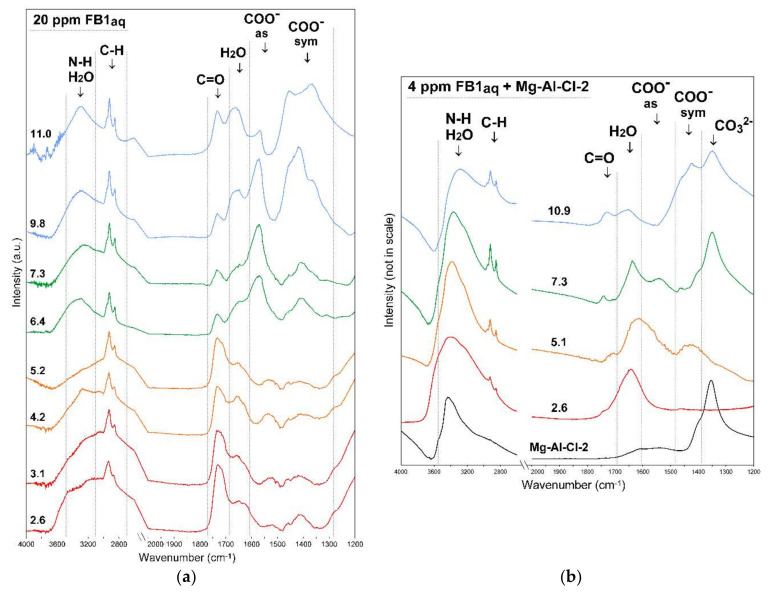
FTIR spectra of: (**a**) FB1 versus pH (C_FB1_ 20 ppm) and (**b**) FB1-treated Mg-Al-Cl-2 sample versus pH (C_FB1_ 4 ppm). The values on the graphs indicate equilibrium pH.

The protonation/deprotonation behavior of pure FB1 was followed with ATR-FTIR by analyzing its 20 ppm aqueous solution in the range from 2.6–11.0 (Figure 11a). In the FB1 spectrum, the following regions can be distinguished and assigned to molecular vibrations: 3500–3100 cm^−1^: N-H and H_2_O stretching, 3100–2700 cm^−1^: C-H stretching, 1770–1680 cm^−1^: C=O stretching, 1680–1600 cm^−1^: H_2_O bending, 1680–1490 cm^−1^: COO^−^_as_ stretching, and 1490–1280 cm^−1^: COO^−^_sym_ stretching. The most visible changes upon pH increase are visible in the regions assigned to COO^−^ groups where bands with maxima at ~1570 cm^−1^ and 1410 cm^−1^ appear (Figure 11a). This confirms deprotonation phenomena of FB1 which is clear at pH_eq_ 6.4. However, it should be underlined that according to computer modeling, the FB1 molecule can be negatively charged already at pH 4 as it undergoes gradual deprotonation, e.g., in EDTA and BTCA compounds [31]. These types of molecules have few pKa values corresponding to several carboxyl groups. The pH effect on the FB1 structure is also visible in the N-H stretching region where at high pH a more symmetric band with maximum at 3300 cm^−1^ can be observed and assigned to free NH_2_. In turn, in acidic conditions where the protonation of NH_2_ occurs the bands in this region become broad with maxima at 3450 and 3150 cm^−1^. The C-H stretching bands were only slightly affected by the pH and these changes may be connected with the conformational changes of FB1. Previous molecular modeling studies conducted in vacuo showed that the backbone carbon chain may fold to a certain degree and FB1 creates a cage-like structure [29,32]. However, later investigations on FB1 behavior in aqueous solutions proved that stable folded conformation are unlikely due to the shielding properties of water molecules [28]. Thus, most probably the FB1 exists in a relatively extended structural state in an aqueous solution.

In order to investigate the changes of FB1 removal efficiency versus pH, the Mg-Al-Cl-2 sample was reacted with FB1 and afterwards the FTIR spectra were recorded (Figure 11b). As reported in earlier studies in acidic conditions, the LDH structure becomes unstable and partial dissolution occurs leading to the release of metals belonging to the brucite-like layer [30]. The LDH dissolution was found to reach ~30 wt.% after 24 h of reaction. The FTIR spectrum recorded for pH_eq_ 2.6 proves a partial LDH dissolution with the clear removal of intercalated CO_3_^2−^ due to lack of band present at ~1350 cm^−1^. Therefore, LDH structural instability was responsible for the slightly lower FB1 removal in acidic conditions. On the other hand, in basic conditions (pH_eq_ 10.9), where LDH is stable, the removal efficiency was affected by the competitive adsorption of hydroxide ions. Such an effect is known to drastically reduce the uptake of anionic forms in aqueous solutions [33]. Therefore, although the FB1 was totally deprotonated in these conditions the uptake was not as efficient as it was for neutral pH values. 

The differences in adsorption behavior between pH 5 and 7 may be explained by the analysis of FTIR spectra recorded at pH_eq_ 5.1 and 7.3 (Figure 11b). The most important difference is the lack of CO_3_^2−^ band at ~1350 cm^−1^ for pH_eq_ 5.1 indicating that monodentate carbonates were deintercalated and/or decomposed on LDH surface in this condition. The presence of carbonates is known to hinder the adsorption of anions which is also confirmed in the case of FB1 adsorption. The synthesis of carbonate-free LDHs is possible by, e.g., using inert gas atmosphere and/or decarbonated water. However, due to their high affinity to CO_2_, their use as adsorbents of anions always leads to competitive effects with CO_3_^2−^ formed in the reaction of CO_2_ with H_2_O. This competitive effect subsequently depends on the type of carbonates present in the interlayer which can form monodentate and bidentate coordination and exist as bicarbonates [16,34].

The higher FB1 removal efficiency by low charge LDHs may be connected to the lower number of carbonates and the simultaneous increase of non-polar sites and thus the matching between adsorption domains of LDH with FB1 structure becomes possible. A similar mechanism was reported for high charge smectites in reaction with aflatoxin molecules [6]. The authors suggested that smectite adsorption properties in this case may be improved by charge reduction.

#### 3.3.2. XPS Analysis

**Figure 12:**
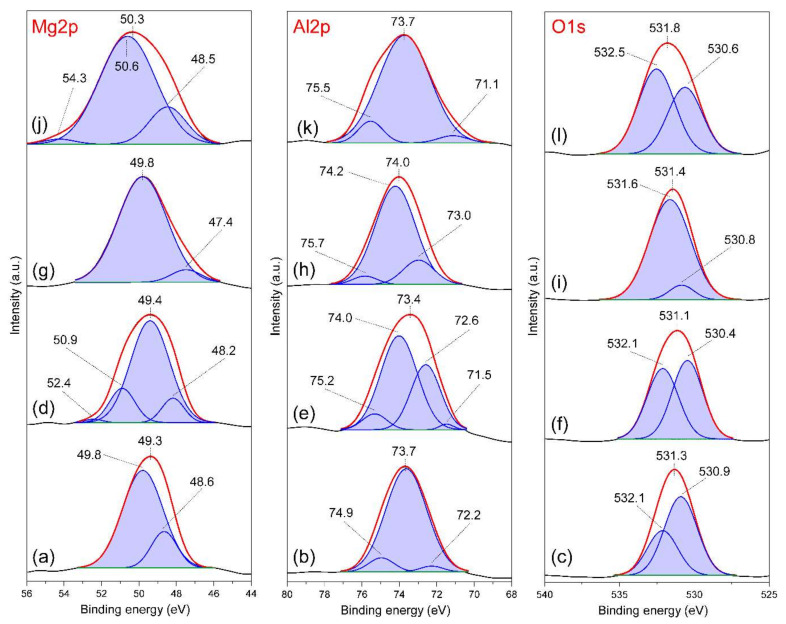
XPS Mg2s, Al2p and O1s spectra of: (**a**–**c**) Mg-Al-Cl-2 sample (pH 7), (**d**–**f**) FB1-treated Mg-Al-Cl-2 sample (pH 7), (**g**–**i**) Mg-Al-Cl-2 sample (pH 5), and (**j**–**l**) FB1-treated Mg-Al-Cl-2 sample (pH 5).

To further investigate the interaction of FB1 with LDH materials, XPS analysis was used to obtain insight into possible changes in the elements’ local environment. The Mg-Al-Cl-2 was selected and Mg2p, Al2p, and O1s spectra were recorded before and after reaction with FB1 at pH 5 and 7 (Figure 12). For the Mg2p spectra, the band with maximum in the range of 49.3–49.8 ppm can be attributed to Mg(OH)_2_ typical for LDH structures (Figure 12a,d,g) [35]. In turn, the maxima of bands corresponding to Al(OH)_3_ building the brucite-like layer are visible in the range of 73.4–73.7 ppm in the Al2p spectra [36]. The O1s spectra show two main components connected to oxygen bonded with Mg (530.4–530.9 ppm) and Al (532.1–532.5 ppm) [36].

Clear changes were visible in the bands’ positions and shapes in the Mg2p, Al2p, and O1s spectra of FB1-treated sample at pH 5 where the adsorption was the most efficient (Figure 12j–l). In particular, the Mg2p spectrum was most affected where the main band was shifted from 49.8 to 50.3 ppm and a new visible component at 50.6 ppm was visible (Figure 12j). This component can be assigned to carboxylate moieties COO^−^ which interact with LDH Mg sites. This agrees with previous reports showing the binding energy of Mg acetate equal to 50.55 ppm in the Mg2p spectra and suggests the electrostatic interaction of FB1 with LDH positively charged sites [37]. This band is less pronounced in the Mg2p spectrum of FB1-treated Mg-Al-Cl-2 sample at pH 7 where the carbonate competition was greater and hindered FB1 adsorption (Figure 12d). This band was not present in the Mg2p spectra of Mg-Al-Cl-2 recorded after equilibration at pH 5 and 7 (Figure 12a,g). The FB1 interaction with Mg sites had a clear impact on the O1s spectra where the main band position was shifted to higher binding energy of 531.8 ppm and the component at 532.5 ppm appeared (Figure 12l). This also confirms the presence of COO^−^ moieties interacting with the LDH surface, in agreement with earlier studies [36,37]. Because the adsorption of FB1 takes place on the brucite-like layers, it results in structural distortions of the metal octahedra and thus the local environment of Al was is altered, as evidenced by the splitting and broadening of bands in the Al2p spectra. However, these changes were not as significant as they were for the Mg.

## 4. Conclusions

The presented study investigated the affinity of chemically different LDH structures to fumonisin B_1_ mycotoxin. The LDH materials (Mg/Al and Mg/Fe) with differing layer charges were synthesized by co-precipitation from both metal nitrates and chlorides. The adsorption experiments showed a clearly greater adsorption capacity for the Mg/Al LDH samples (~0.08–0.15 mol/kg) as compared to the Mg/Fe LDH samples (~0.05–0.09 mol/kg). A significant difference in removal efficiency was not observed between chloride and nitrate intercalated LDH samples. However, a trend of increasing adsorption capacity along with higher Mg/Al ratio (lower layer charge) was noticed. This was attributed to the lower content of bonded carbonates and increase of non-polar sites which led to matching between the adsorption domains of the LDH with the FB1 structure. For all the materials, a visibly greater adsorption was found for pH in the range of 4–5. The FTIR analysis allowed to confirm the negative effect of carbonates which hampered adsorption at pH 7. The highest adsorption capacity measured at pH 5 corresponded to the FB1 content of ~15.8 ± 0.75 wt.%. The kinetic experiment revealed fast adsorption with equilibrium achieved in the first 1–2 min. This suggested the external surface adsorption mechanism, which was confirmed by XRD results, which indicated that no FB1 intercalation occurred. The strong interaction of FB1 and LDH surface was confirmed by XPS technique for the samples equilibrated at different pH values. It was attested by the broadening and splitting of bands in the Mg2p, Al2p, and O1s spectra. In particular, the most significant distortion of Mg sites in the brucite-like layer was observed at pH 5 where the interaction with FB1 carboxylate moieties COO^−^ was confirmed. This research confirmed the high affinity and selectivity of LDH structures towards anionic forms of FB1 mycotoxin. The reported adsorption capacity was much higher than that determined for surfactant-modified zeolitic materials.

## Figures and Tables

**Table 1:** The layered double hydroxide (LDH) materials used in the study with the chemistry of the brucite-like layer.

Chemistry	M(II)/M(III) Assumed Value	M(II)/M(III) Determined Value	Interlayer Anion	Symbol
Mg/Al	2	1.94 ± 0.07	Cl	Mg-Al-Cl-2
Mg/Al	3	2.76 ± 0.07	Cl	Mg-Al-Cl-3
Mg/Al	4	3.24 ± 0.08	Cl	Mg-Al-Cl-4
Mg/Al	6	4.51 ± 0.04	Cl	Mg-Al-Cl-6
Mg/Al	8	5.26 ± 0.04	Cl	Mg-Al-Cl-8
Mg/Al	2	2.09 ± 0.09	NO_3_	Mg-Al-NO_3_-2
Mg/Al	3	2.97 ± 0.06	NO_3_	Mg-Al-NO_3_-3
Mg/Al	4	3.56 ± 0.06	NO_3_	Mg-Al-NO_3_-4
Mg/Fe	2	1.72 ± 0.01	Cl	Mg-Fe-Cl-2
Mg/Fe	3	2.48 ± 0.01	Cl	Mg-Fe-Cl-3
Mg/Fe	4	3.32 ± 0.02	Cl	Mg-Fe-Cl-4
Mg/Fe	2	1.88 ± 0.01	NO_3_	Mg-Fe-NO_3_-2
Mg/Fe	3	2.58 ± 0.02	NO_3_	Mg-Fe-NO_3_-3
Mg/Fe	4	3.53 ± 0.03	NO_3_	Mg-Fe-NO_3_-4

## Supplementary Materials

The following are available online at https://www.mdpi.com/1996-1944/13/19/4344/s1, Figure S1: Particle size distribution of the raw LDH samples (y axis—Volume (% a.u.)—one spacing on the y axis corresponds to 4%).
