# Supplementary material for: Fumonisin B1 Interaction with Mg-Al and Mg-Fe Layered Double Hydroxides: Removal Efficiency and Mechanisms
Source: Materials (Basel). 2020 Sep 29;13(19):4344. doi: 10.3390/ma13194344 (PMC7579089; doi:10.3390/ma13194344)
Supplement: Supplementary file 1 [file materials-13-04344-s001.pdf]

Supplementary Information

Fumonisin B_1 Interaction with Mg-Al and Mg-Fe Layered Double Hydroxides: Removal Efficiency and Mechanisms

Jakub Matusik [1,*] and Youjun Deng [2]

1. Faculty of Geology, Geophysics and Environmental Protection; Department of Mineralogy, Petrography and Geochemistry, AGH University of Science and Technology, al. Mickiewicza 30, 30-059 Krakow, Poland
2. Department of Soil and Crop Sciences, Texas A&M University, College Station, TX 77843-2474, USA; yjd@tamu.edu

* Correspondence: jmatusik@agh.edu.pl; Tel.: +48-126175233s

Received: 8 September 2020; Accepted: 28 September 2020; Published: date

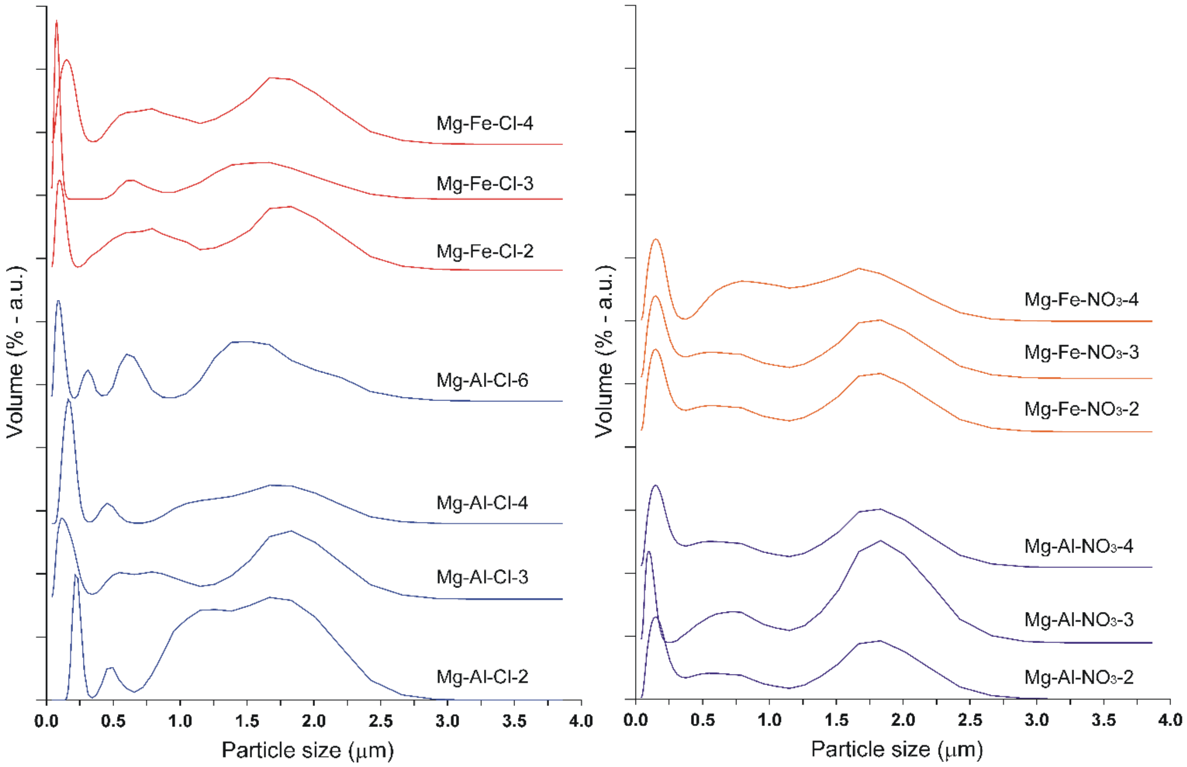

Figure S1. Particle size distribution of the raw LDH samples (y axis—Volume (% a.u.)—one spacing on the y axis corresponds to 4%).
